# Steroid-resistant nephrotic syndrome in infants caused by a novel compound heterozygous mutation of the *NUP93*: A CARE case report

**DOI:** 10.1097/MD.0000000000024627

**Published:** 2021-02-12

**Authors:** Bo Zhao, Ji-Yu Chen, Ya-Bin Liao, Yan-Fang Li, Xue-Mei Jiang, Xin Bi, Mi-Feng Yang, Li Li, Jing-Jing Cui

**Affiliations:** a Department of Nephrology and Rheumatology, Kunming Children's Hospital, Kunming; b Dali University, Dali; c Kunming Key Laboratory of Children Infection and Immunity, Yunnan Key Laboratory of Children's Major Disease Research, Yunnan Medical Center for Pediatric Diseases, Yunnan Institute of Pediatrics, Kunming Children's Hospital, Kunming, Yunnan, China.

**Keywords:** Chinese infant, genetics, *NUP93*, steroid-resistant nephrotic syndrome

## Abstract

**Rationale:**

Steroid-resistant nephrotic syndrome (SRNS) is a special kidney disease. SRNS is characterized by steroid-resistant, clinical variability, and genetic heterogeneity. Patients with SRNS often may eventually need renal transplantation.

**Patient concerns:**

A 10-month-old Chinese male infant presented with oliguria, renal dysfunction, hypertension, and anemia.

**Diagnoses:**

Combined with clinical manifestations, laboratory testing and sequencing results, the patient was diagnosed as SRNS.

**Interventions:**

Combined intravenous methylprednisolone and cefoperazone sulbactam did not improve the patient's condition. Thus, SRNS associated with hereditary nephrotic syndrome was strongly suspected. Genetic testing for hereditary renal disease of the patient revealed 2 novel heterozygous mutations in the Nucleoporin 93 (*NUP93*) gene, which were predicted pathogenic and harmful by bioinformatic softwares of SIFT, PolyPhen_2 and REVEL.

**Outcomes:**

As general physical health deterioration and renal dysfunction, the patient died of a severe infection.

**Lessons:**

The novel *NUP93* heterozygous mutations identified in the current study broadened the genetic spectrum of SRNS and further deepened our insight into pathogenic mutations of *NUP93* to improve disease diagnosis.

## 1 Introduction

Nephrotic syndrome (NS) is a clinical syndrome caused by increased permeability of the glomerular filtration membrane to plasma proteins, resulting in the loss of large quantities of plasma proteins from the urine. This causes a series of pathophysiologic changes characterized by massive proteinuria, hypoproteinemia, hyperlipidemia, and edema. The annual incidence of NS among children <16 years of age in the United States has been reported to be around 2 to 7 per 100,000. Sixteen of every 100,000 children are diagnosed with NS, making NS one of the commonest renal diseases in children.^[1]^ NS can be classified into steroid-sensitive NS and steroid-resistant NS (SRNS) based on the response of NS patients to corticosteroid therapy.^[2]^ Most children with NS respond well to steroids, but around 10% to 20% are diagnosed with SRNS. SRNS is a renal disease presenting with clinical variability and genetic heterogeneity. A higher frequency of genetic variations has been reported in early-onset SRNS than steroid-sensitive NS. Currently, both treatment and prognosis of this disease depend on the renal pathologic diagnosis. Unfortunately, there are still no effective treatment regimens for SRNS, and approximately 50% of patients who have developed SRNS in childhood exhibit focal segmental glomerulosclerosis (FSGS) and are at high risk of developing end-stage renal disease (ESRD). Renal transplantation may eventually be required.^[3]^

To date, at least 50 genes have been shown to be associated with SRNS when mutated.^[4]^ It has been reported that approximately 29.5% of families with a member diagnosed with SRNS and <25 years of age are caused by a single gene mutation. Among the mutations, the *NPHS1*, *NPHS2*, and *WT1* are the most common causes of SRNS.^[5]^ It has previously been reported that among 160 SRNS families, a Nucleoporin 93 (*NUP93*) mutation was responsible for the pathogenesis of 3 families with hereditary renal disease.^[6]^ Based on high-throughput exon sequencing in >1800 SRNS families, *NUP93* variations, including compound heterozygous and missense variants, were identified to cause this disease in 3 families.^[7]^ In addition, a case of SRNS caused by *NUP93* a gene mutation was recently reported by Japanese scholars.^[23]^ A case of SRNS caused by a novel compound heterozygous mutation of *NUP93*, which has not been previously reported, is described herein.

## 2 Case report

A 7-month-old Chinese male infant with edema and proteinuria received symptomatic treatment at a local hospital. At 8 months of age, he was diagnosed clinically with nephritic NS due to edema, proteinuria, hypoproteinemia, microscopic hematuria, and hypertension, and thus received prednisone at a dose of 2 mg/kg/d for 8 weeks in Thailand. The edema was not relieved and the proteinuria did not resolve. At 10 months of age, the patient was admitted to our hospital due to cough, shortness of breath, oliguria, renal dysfunction, hypertension, and anemia. The infant was the third child of the family and delivered with a birth weight of 2400 g. His family has no history of renal disease. Peripheral blood samples of the patient and his parents were collected for gene sequencing.

The physical examination findings of the infant at the time of admission were as follows: body temperature, 36.3°C; heart rate, 126 bpm; respiratory rate, 36/min; blood pressure, 152/103 mm Hg; weight, 9.5 kg; an appearance of anemia; anasarca; abdominal distension; no systemic rashes or papulovesicles; and moist rales auscultated in the lungs bilaterally.

The biochemical indices of the patient are summarized in Table 1. The albumin and globulin levels were decreased, the creatinine, urea, uric acid, lactate dehydrogenase, total cholesterol, triglycerides, and complement C4 levels were increased, and the serum complement C3 level was normal. All the patient's immune-related antibodies were negative, which included antinuclear antibodies, anti-Sm antibodies, antineutrophil cytoplasmic antibodies, etc. the urine analysis revealed 4+ proteinuria, and there were 13 red blood cell/high power field. The hematologic examination revealed hypochromic microcytic anemia (hemoglobin, 69 g/L; mean corpuscular volume, 79.6 fL; mean corpuscular hemoglobin, 23.7 pg; and mean corpuscular hemoglobin concentration, 305 g/L), while the platelet count and the serum C-reactive protein level were normal.

**Table 1:** Laboratory examination results of the patient.

Items	Results	References
Albumin	20.2 g/L	35 g/L–50 g/L
Globulin	21.9 g/L	20 g/L–40 g/L
Creatinine	239.75 μmol/L	27 μmol/L–62 μmol/L
Urea nitrogen	16.42 μmol/L	1.8 μmol/L–6.4 μmol/L
Lactic dehydrogenase	499 μ/L	67 μ/L–394 μ/L
Total cholesterol	9.12 mmol/L	3.12 mmol/L–5.2 mmol/L
Triglyceride	5.4 mmol/L	0.8 mmol/L–1.8 mmol/L
Complement C3	0.85 g/L	0.8 g/L–1.5 g/L
Complement C4	0.48 g/L	0.12 g/L–0.4 g/L
Urinary protein	4+	negative
Urine red cell	13/HPF	0-3/HPF

Relevant imaging examinations were obtained. Abdominal color Doppler ultrasonography showed a moderate peritoneal effusion, and a chest X-ray revealed exudation in both lungs and a pleural effusion.

**Figure 1:**
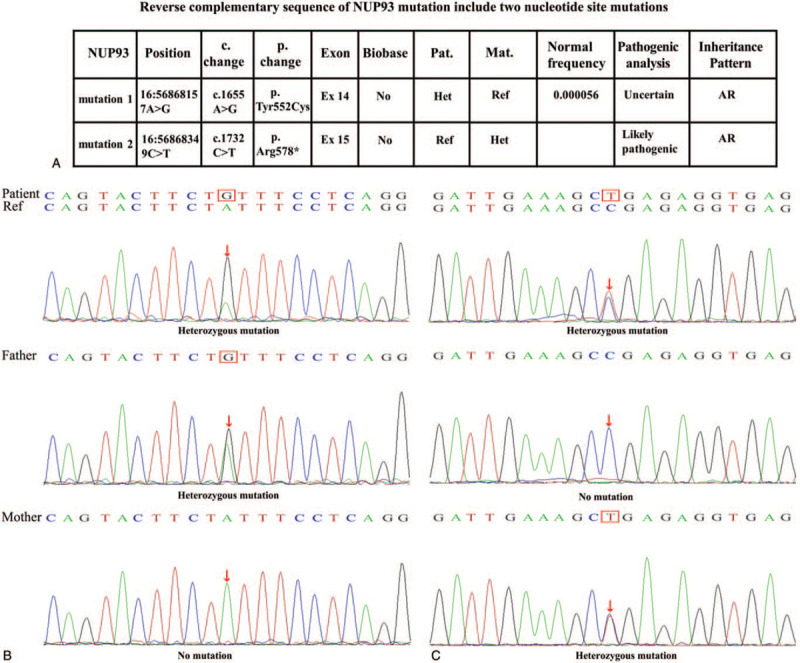
Genotype and conservation of the patient's mutations in *NUP93*: A. Table with Chromosome position, c. change, p. change, exon number, nomal frequency, pathogenic analysis and inheritance pattern in our patient. B. Sanger tracing for the patient, the mother and father for each allele.

Based on the clinical manifestations and the results of the laboratory testing, hereditary NS was strongly suspected. Therefore, all exons of 506 genes associated with urinary system diseases were analyzed using next generation sequencing. The infant harbored 2 novel heterozygous mutations in *NUP93* (NM_014669), and were subsequently confirmed by Sanger sequencing: a paternal missense variant c.1655A>G (p.Tyr552Cys) and a maternal nonsense variant c.1732C>T (p. Arg578∗) (Fig. 1). After searching the mutation records of normal population in 1000 Genome Project, NHLBI Exome Sequencing Project (ESP6500), Exome Aggregation Consortium (ExAC_ALL), and Exome Aggregation Consortium East Asian (ExAC_EAS), a very low frequency of c.1655A>G (p.Tyr552Cys) and no information of the frequency of c.1732C>T (p. Arg578∗) in normal population were found. No related reports have been found in the Human Gene Mutation Database Professional database. The patient was treated with intravenous methylprednisolone (2 mg/kg/d) and cefoperazone-sulbactam (100 mg/kg/d) for 1 week did not improve the patient's condition. The clinical disease progressed rapidly and was accompanied with anemia and renal dysfunction. The patient died of a severe infection.

The study was approved by the Ethics Committee at the Kunming Children's Hospital, Yunnan, China (2016-03-022-K01). All of the patients provided written informed consent for publication of this case report and accompanying images.

## 3 Discussion

NS is a clinical syndrome caused by damage to the glomerular filtration barrier. NS is a common glomerular disease in children, the incidence and responsiveness to steroids of which varies with ethnicity.^[8]^ In our case, the infant was diagnosed with SRNS based on the clinical manifestations and results of laboratory testing.

**Figure 2:**
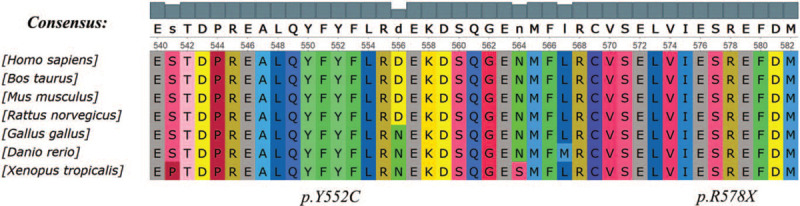
Conservative analysis of amino acid 552 and amino acid 578 of *NUP93.*

**Figure 3:**
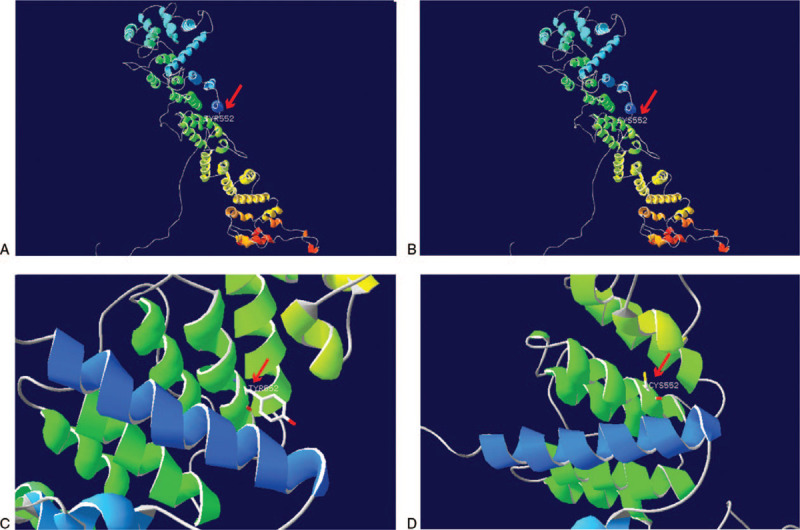
Structural modeling of the wild type and mutant *NUP93* mutation (Y552C): The structure modeling is based on the Electron Microscopy determined coordinates of the composite structure of the inner ring of the human nuclear pore complex using SWISS-MODEL (SMTL ID: 5ijn.1). A and C: A structural model of the wild NPC protein NUP93 with 100% sequence identity is demonstrated. B and D: A structural alteration of the mutant NPC protein NUP93 with 99.88% sequence identity is shown. The locations of the NUP93 mutation (Y552C) mutations (red arrows) are indicated. The benzene ring structure (red arrows) was disrupted when tyrosine was replaced by cysteine as a result of c. 1655A>G mutation.

A growing body of literature has shown that 29.8% of SRNS cases are caused by single gene mutations in various genes, such as *NPHS2*, *NPSH1*,^[9]^*WT1*, *LAMB2*, and *PLCE1*.^[10,11]^ In our patient, the genetic analysis revealed a compound heterozygous variations in *NUP93*, one is a novel paternal missense variant in exon 14, c.1655A>G (p.Tyr552Cys), with a frequency of 0.00006 in normal population in the gnomAD_genome database (http://gnomad.broadinstitute.org/). This site has not been reported before. The effect of this mutation on the protein function was predicted pathogenic and harmful by bioinformatic softwares of SIFT, PolyPhen_2, and REVEL. Another is a maternal nonsense variant in exon 15, c.1732C>T (p. Arg578∗), which was rated to be likely pathogenic according to the American College of Medical Genetics and Genomics guidelines. No information of the frequency of this site in normal population was found. The 2 sites of NUP93 (NM_014669): c.1655A>G (p.Tyr552Cys)/c.1732C>T (p. Arg578∗) are highly conserved sequence across multiple species (Fig. 2). When modeled by SWISS-MODEL, one key benzene ring structure was found to be absent in the three-dimensional structural model of the Nuclear Pore Complex Protein *NUP93* (Fig. 3). A mutation in the *NUP93* is one of the very rare causes of hereditary SRNS.^[12,13]^

*NUP93* is a widely expressed gene located on chromosome 16q13 and includes 22 exons and 21 introns that encodes a highly conserved nucleoporin. Thirty different nucleoporins (NUPs) will form a nuclear pore complex (NPC), which is a macromolecular (approximately 50 MDa) protein assembly embedded in the nuclear membrane.^[14]^ NPC, composed of multiple copies of NUPs,^[15]^ mediates bidirectional nucleo-cytoplasmic transport of proteins, RNA, and ribonucleoprotein particles. There are 2 major supporting modules in the NUP that constitute a symmetric structure spanning from the inner nuclear membrane to the outer nuclear membrane, thus forming a central pathway.^[16]^ These supporting modules coordinate structures of the *NUP93* complex by interacting directly or indirectly with other NUPs (*NUP53*, *NUP155*, *NUP188*, and *NUP205*) at the C-terminal α-helical domain of *NUP93*, and this complex regulates the permeability barrier of pores.^[17]^ Thus, *NUP93* plays a crucial role in organizing nuclear pores in NPC; the aberrant *NUP93* can disrupt the NUP assembly, and in turn induce dysfunction of nucleo-cytoplasmic transport.^[18,19]^ In addition, a recent study suggested that mutation of *NUP93* inhibits the proliferation of podocytes and possibly cause FSGS,^[20]^ but the underlying molecular mechanism has not been established.

*NUP93* mutation-related SRNS has been reported in 11 separate SRNS families (Table 2), with different ethnicities, ages of onset, and different mutation sites and patterns. Our patient and 10 previously reported cases all presented with SRNS. Eight out of the 10 reported cases was subjected to renal biopsy and their renal pathology was all determined as FSGS. Of the 10 reported cases, 3 had extra-renal manifestations and 7 had not. Their average ages of the disease onset and of developing ESRD were 3.5 years and 5.4 years, respectively. Of these 10 children, 8 were heterozygous mutations and 2 were homozygous mutations, and their mutation sites were distributed in exons 11 to 14, 16 to 19, 7, and 21. In contrast, the age of the disease onset in our child was smaller than that of the previously reported cases, with an age of only 0.6 years, and he was only 0.8 years old when he developing ESRD. In addition, the extra-renal symptoms were absent in our patient. Importantly, he was found to harbor *NUP93* mutations in exons 14 and 15, constituting heterozygous mutation.

**Table 2:** Description of genetic diagnosis of *NUP93* gene mutations in 11 different families with SRNS, gene locus detected with variations, and some clinical features.

Family/individual	Nucleotide change	Amino acid change	Zygosity	Exon	Gender	Ancestry	Onset/ ESRD year	Proteinuria	Haematuria	Edema	Hypertension	Extrarenal manifestation/DS	Renal biopsy	Therapy/response	RTX	Ref
No.1	c.1162 C>T /c.1772G>T	p.A388T /p.G591V	Het	Ex11/16	Female	Serbian	6 yr/6 years	Y	Y	Y	UK	N	FSGS	RTX /HK	Y	^[20]^
No.2	c.1326delG /c.1772G>T	p.L442A /p.G591V	Het	Ex12/16	Female	German	3 yr/3 yr	Y	Y	Y	UK	N	FSGS	CSA/PR	Y	^[20]^
No.3	c.1537+1G>A /c.1772G>T	del exon13/p.G591V	Het	Ex13/16	Female	German	3 yr/4 yr	Y	Y	Y	UK	MG-syndrome	FSGS	SRNS	Y	^[20]^
No.4	c.1772G>T	p.G591V	Hom	Ex16	Male	Turkish	3 yr/11 yr	Y	Y	Y	UK	N	FSGS	SRNS	N	^[20]^
No.5	c.1886A>G	p.T629C	Hom	Ex17	Male	Turkish	1 yr/ 1 yr	Y	Y	Y	UK	N	FSGS	SRNS	Y	^[20]^
No.6	c.1772G>T /c.1298delA	p.G591V/ p.D433A	Het	Ex16/12	Female	Czsk	3 yr/ 3.5 yr	Y	UK	Y	UK	N	FSGS	ESRD	Y	^[21]^
No.7	c.1772G>T/c.1916 T>C	p.G591V/ p.L639P	Het	Ex16/18	Male	Czsk	1.8 yr/ 2.9 yr	Y	UK	Y	UK	N	FSGS	CSA/ESRD	Y	^[21]^
No.8	c.2084T>C/c.2267T>C	p.L695S/ p.L756S	Het	Ex19/21	Male	White	6.1 yr/ 12 yr	Y	UK	UK	UK	UK	UK	SRNS	UK	^[22]^
No.9	c.1573C>T/c.1886A>G	p.R525W/ p.Y629C	Het	Ex14/17	Female	Japanese	4 yr/ 6 yr	Y	Y	Y	Y	RA	FSGS	SRNS	Y	^[23]^
No.10	c.575A>G/c. 1605C>G	p.T192C/ p.Tyr535Ter	Het	Ex7/14	Female	American	5 yr/ 5 yr	Y	Y	Y	UK	CM, AF	UK	SRNS	Y	^[13]^
New report	c.1655A>G/c.1732C>T	p.T192C/ p.Tyr535Ter	Het	Ex14/15	Male	Chinese	0.6 yr/ 0.8 yr	Y	Y	Y	Y	N	UK	SRNS	N	[^∗^]

AF = autistic features, CM = cardiomyopathy, CSA = cyclosporin A, CZSK = Czech and Slovak, DS = dysmorphic-syndrome, ESRD = end stage renal disease, Ex = Exon, HK = hyperechogenic kidneys, MG = Marcus-Gunn, N = No, PR = partial response, RA = rheumatoid arthritis, Ref = reference, RTX = renal transplantation, UK = unknown, Y = Yes.

∗ The present case.

Taken together, the novel mutation of the *NUP93* detected in this patient might cause SRNS, but this mutation resulted in the functional alteration of the corresponding protein has only been validated in protein function prediction and 3D protein structure models prediction. In clinical practice, gene sequencing should be performed for infantile-onset SRNS to determine whether the disease is congenital, thereby accurate and adequate treatment methods can be used to improve the outcome of this disease.

## Author contributions

**Conceptualization:** Bo Zhao, Li Li.

**Formal analysis:** Ji-Yu Chen, Jing-Jing Cui.

**Methodology:** Ya-Bin Liao, Yan-Fang Li.

**Resources:** Yan-Fang Li, Xue-Mei Jiang.

**Software:** Ji-Yu Chen.

**Supervision:** Xue-Mei Jiang, Xin Bi, Mi-Feng Yang.

**Validation:** Xin Bi, Mi-Feng Yang.

**Writing – original draft:** Bo Zhao, Ji-Yu Chen.

**Writing – review & editing:** Ya-Bin Liao, Li Li, Jing-Jing Cui, Ji-Yu Chen.

## Abbreviations

Abbreviations: ESRD = end-stage renal disease, FSGS = focal segmental glomerulosclerosis, NPC = nuclear pore complex, NS = nephrotic syndrome, NUP93 = Nucleoporin 93, NUPs = nucleoporins, SRNS = steroid-resistant nephrotic syndrome, SSNS = steroid-sensitive nephrotic syndrome.
